# Heat Sensing Receptor TRPV1 Is a Mediator of Thermotaxis in Human Spermatozoa

**DOI:** 10.1371/journal.pone.0167622

**Published:** 2016-12-16

**Authors:** Luca De Toni, Andrea Garolla, Massimo Menegazzo, Sabina Magagna, Andrea Di Nisio, Iva Šabović, Maria Santa Rocca, Valentina Scattolini, Andrea Filippi, Carlo Foresta

**Affiliations:** 1 Department of Medicine, Unit of Andrology and Reproductive Medicine, University of Padova, Via Giustiniani, Padova, Italy; 2 IRCCS-Istituto Oncologico Veneto [IOV], Via Gattamelata, Padova, Italy; 3 Department of Medicine, University of Padova, Via Giustiniani, Padova, Italy; 4 Venetian Institute of Molecular Medicine, Via Orus 2, Padova, Italy; 5 Department of Physics and Astronomy “G. Galilei”, University of Padova, Via Marzolo 8, Padova, Italy; Duke University School of Medicine, UNITED STATES

## Abstract

The molecular bases of sperm thermotaxis, the temperature-oriented cell motility, are currently under investigation. Thermal perception relies on a subclass of the transient receptor potential [TRP] channels, whose member TRPV1 is acknowledged as the heat sensing receptor. Here we investigated the involvement of TRPV1 in human sperm thermotaxis. We obtained semen samples from 16 normozoospermic subjects attending an infertility survey programme, testis biopsies from 6 patients with testicular germ cell cancer and testis fine needle aspirates from 6 patients with obstructive azoospermia undergoing assisted reproductive technologies. Expression of *TRPV1* mRNA was assessed by RT-PCR. Protein expression of TRPV1 was determined by western blot, flow cytometry and immunofluorescence. Sperm motility was assessed by Sperm Class Analyser. Acrosome reaction, apoptosis and intracellular-Ca^2+^ content were assessed by flow cytometry. We found that *TRPV1* mRNA and protein were highly expressed in the testis, in both Sertoli cells and germ-line cells. Moreover, compared to no-gradient controls at 31°C or 37°C (Ctrl 31°C and Ctrl 37°C respectively), sperm migration towards a temperature gradient of 31–37°C (T gradient) in non-capacitated conditions selected a higher number of cells (14,9 ± 4,2×10^6^ cells T gradient *vs* 5,1± 0,3×10^6^ cells Ctrl 31°C and 5,71±0,74×10^6^ cells Ctrl 37°C; P = 0,039). Capacitation amplified the migrating capability towards the T gradient. Sperms migrated towards the T gradient showed enriched levels of both TRPV1 protein and mRNA. In addition, sperm cells were able to migrate toward a gradient of capsaicin, a specific agonist of TRPV1, whilst capsazepine, a specific agonist of TRPV1, blocked this effect. Finally, capsazepine severely blunted migration towards T gradient without abolishing. These results suggest that TRPV1 may represent a facilitating mediator of sperm thermotaxis.

## Introduction

The encounter between the sperm cell and the oocyte within the female reproductive tract leads to a cascade of events, known as fertilization, consisting of sperm penetration, sperm–oocyte fusion, and zygote division. The chance of this cell-to-cell contact is very narrow and results from a number of highly regulated attracting mechanisms that are believed to guide spermatozoa along the oviduct [1]. Indeed, a redundancy of mid-low molecular weight molecules gained value as chemoattractants for sperm cells. Steroids, like progesterone [2], to chemokines, either released by the cumulus oophorus or the oocyte [3 4], display chemoattractant properties toward spermatozoa. Even OR1D2, an olfactory receptor normally expressed in human olfactory epithelium, has been acknowledged to play a critical role in the drive of sperm-oriented motility [5]. However, chemotaxis itself may not be a sufficient drive for spermatozoa, since the peristaltic movements of the oviduct are expected to shuffle tubal fluid and prevent the formation of a long-range chemoattractant gradient *in vivo* [6]. This would restrict the role of chemotaxis to a short distance range from the oocyte [7]. To this regard, earlier studies found that a difference in temperature exists between the site of sperm deposition (cooler) and the site of possible fertilization (warmer) at ovulation. In particular, a temperature difference of 0,7–2°C has been detected between the isthmus and the isthmic-ampullary junction in pigs and rabbits [6, 8]. Unfortunately, there are no published measurements in humans [6]. Mammalian spermatozoa have the capacity to sense these small temperature differences that act as a major drive for the guide of male gametes from the reservoir towards the warmer fertilization site, a process named thermotaxis [9]. Moreover, the exposure to increasing temperature has a major impact on the membrane structure and the gain of fluidity which is prodromal to sperm capacitation and acrosome reaction [10]. *In vivo*, thermotaxis is likely to be complementary to chemotaxis, each mechanism being functional in a region where the other mechanism is ineffective. It has been hypothesized that, *in vivo*, spermatozoa are first guided by thermotaxis from the reservoir towards the warmer fertilization site, then, at close proximity to the oocyte and within the cumulus mass, the guidance is likely carried out by chemotaxis [6].

The molecular bases of thermotaxis are currently under investigation [11, 12]. As evidenced by recent studies, thermal sensation relies on a subclass of the transient receptor potential (TRP) channels. More than 30 members of mammalian TRP channel family have been characterized and can be subdivided into seven main classes according to their sequence homology. Only six TRPs are recognized as temperature sensitive-TRPs since they are expressed in primary somatosensory neurons and are activated at specific temperatures in the range from noxious heat to painful cold (reviewed in [13]). A heat-sensitive non-selective ion channel, supposed to be responsible for much of the heat sensitivity of primary sensory neurons, has been cloned and named transient receptor potential vanilloid receptor 1 (TRPV1) because it is specifically activated by vanilloids such as capsaicin, a pungent chemical found in hot chilli peppers [14]. Although initially controversial, it is now firmly established that TRPV1 is expressed in non-neuronal tissues, such as dermis, blood vessels, where it could play a wide variety of physiological functions [15]. To this regard, the expression of TRPV1 by human sperm cells and its involvement in calcium trafficking has been recently described [16–18].

In this study, we investigated the expression of TRPV1 in different cell populations of human testis. Moreover, we evaluated the involvement of TRPV1 in the modification of sperm functions subtending thermotaxis.

## Materials and Methods

### Chemicals

Rabbit polyclonal anti-TRPV1 antibody and control peptide antigen were purchased from Alomone Labs (Jerusalem, Israel). FITC-conjugated anti-rabbit IgG, normal donkey serum, capsaicin (CPS), capsazepine (CPZ) and acridine orange-Zinc chloride salt were purchased from Millipore (Merck Group, Vimodrone, Milano, Italy). Mouse monoclonal FITC-conjugated anti-human CD46 antibody was purchased from BD-Biosciences (Milano, Italy). Calcium Orange^TM^ AM was purchased from Thermo Fisher Scientific (Milano, Italy). Sperm washing medium (SWM) was purchased by Irvine Scientific (Santa Ana, CA, USA).

### Semen samples

All subjects provided written informed consent for the study, which had been previously approved by the local Ethical Committee for Clinical Trials of the Padova University Hospital (protocol n. 2208P). The investigation was performed according to the principles of the Declaration of Helsinki. Semen was obtained from 16 normozoospermic healthy donors, attending the University Andrology Unit as participants in an infertility survey programme (mean age 28,1 ± 5,2 years).

In order to reduce any inter-subject variability, patients underwent to 4 separate sessions of semen donation by masturbation, taking care to maintain at least 3 days of sexual abstinence between each donation. Samples obtained from each session were used respectively for thermotaxis, pharmacological modulation of TRPV1, calcium imaging and pharmacological modulation of TRPV1 during thermotaxis as detailed below. Samples were examined according to the WHO criteria [19]. Capacitation was performed as described elsewhere with slight modifications [20]. Sperm motility parameters such as curvilinear velocity (VLC), straight-line velocity (VSL), average path velocity (VAP), amplitude of lateral head displacement (ALH), linearity of a curvilinear path (LIN, defined as VSL/VLC), straightness (STR, defined as VSL/VAP), wobble (WOB, defined as VAP/VCL), beat-cross frequency (BCF), hypermotile sperm fraction (Hyper), were assessed on cells retrieved from the recovery chamber of thermotaxis device (see below), the with the Sperm Class Analyser (SCA, Microptic S.L., Barcelona, Spain) as previously described [4].

### Biopsies and testis fine needle aspiration

Paraffin embedded sections of testis biopsies were obtained from 6 patients affected by testicular germ cell tumours (kindly gifted by Dr. Poletti, Histology and Pathological Anatomy Unit, Bassano del Grappa Hospital, Italy). Specimens from cancer patients were derived from the healthy testicular tissue surrounding cancer, featured by tubules with normal spermatogenesis, as assessed by previous histological evaluation.

6 patients, featured by idiopathic obstructive azoospermia and free from any mutation of the CFTR gene [21], underwent testicular fine needle aspiration (FNA) aimed to sperm retrieval and cryopreservation for subsequent assisted reproductive technologies as previously described [22].

### Sperm thermotaxis

Sperm thermotaxis was assessed as previously described with slight modifications [23], by the use of a device (40 mm length) constituted by two chambers, one for loading and one for recovery, filled with SWM. A temperature gradient (successively “T gradient”) was created by placing the loading and the recovery chambers onto two thermostatic plates respectively at 31°C and 37°C, achieving a linear temperature gradient of 1,5°C/cm, comparable to what reported in similar studies [12]. As controls, the same procedure was repeated by maintaining both chambers at the same temperature of either 31°C (Ctrl 31°C) or 37°C (Ctrl 37°C) with no gradient along the tube.

### Pharmacological modulation of TRPV1

Experiments of pharmacological modulation of TRPV1 were performed in the same device used for thermotaxis maintained at 31°C unless otherwise stated. The recovery chamber was filled with CPS, at concentrations ranging from 2,5 to 160 μM, that was allowed to diffuse towards the loading chamber for a standard time of 5 minutes. TRPV1 inhibition was performed by incubating sperm cells with either CPZ, at concentrations ranging from 2,5 to 160 μM, or rabbit polyclonal anti-TRPV1 antibody at the final concentration of 2,5 μM as previously described [24, 25]. Cells were successively washed and then allowed to migrate towards a CPS gradient as described.

## Immunofluorescence

Expression of TRPV1 was evaluated on both paraffin-embedded sections of testis, smeared testicular FNA and sperm cells retrieved from migration experiments by immunofluorescence (IF) as previously described [26].

### Western Blot

Expression of TRPV1 was evaluated by western blot analysis, on FNA specimens from patients with idiopathic obstructive azoospermia, ejaculated spermatozoa, as previously described [27]. Isolated sperm-plasma membrane were also obtained as previously described [28]. Briefly, cell pellet was suspended in hypotonic buffer and homogenized. Intact cells and nuclei were pelleted at 1000 × g for 10 min. The postnuclear supernatant was centrifuged at 5000×g for 20 min to obtain a light membrane suspension that was subsequently suspended in 100 mM Na2CO3 (pH 11.6) in 1 M NaCl and further ultracentrifuged at 200 000 × g for 2 h. to obtain a pellet consisting of washed membranes.

### Flow Cytometry and Calcium Imaging

The functional status of sperm cells was assessed by flow cytometry as previously described [4]. Briefly, sperm TRPV1 levels were evaluated by incubation with anti-TRPV1 antibody (1:50 in PBS) for 30 min at 4°C. Acrosome reaction was measured through incubation with anti-human CD46 antibody (1:50 in PBS, BD Biosciences) for 30 min at 4°C. Intracellular calcium levels were assessed through incubation with 10 μM Calcium Orange^TM^ AM (Thermo Fisher Scientific) for 30 minutes at room temperature. Apoptosis was assessed through Apoptosis Detection Kit (BD Biosciences San Jose, CA, USA) according to the manufacturer’s instructions. The gating strategy consisted of a morphological scatter plot where only viable PI-negative cells were considered for further analysis (S1A Fig).

Intracellular calcium imaging was performed as previously described [29]. Briefly, sperm cells were loaded with 10 μM Fluo-4 AM (ThermoFisher) and Pluronic F–127 (0.1% w/v) (Sigma-Aldrich) and then analyzed with a two-photon a modular multiphoton microscope (Bergamo II, Thorlabs) under varying stimuli conditions. Verapamil was added at the final concentration of 50μM in order to exclude the possible involvement of voltage gated Ca^2+^ channels. Image analysis was performed by the use of in-house developed software in Matlab environment (Release 14, The MathWorks, Inc., Natick, MA, USA).

### RNA Extraction, cDNA Synthesis and Real Time-PCR

*TRPV1* mRNA expression in FNA specimens from patients with idiopathic obstructive azoospermia and ejaculated spermatozoa was performed by RT-PCR as previously described [27]. Primers for TRPV1 were: forward 5’-AGCAGTGCCTTCTTCATCCT -3’ and reverse 5’- TGGATGGAGTGGAAGCATGT-3’ (186 bp). As housekeeping gene we used GAPDH (forward 5’-AAGGTGAAGGTCGGAGTCAA-3’ and reverse 5’-AATGAAGGGGTCATTGATGG-3’). Relative quantification was performed, using ΔΔCt method. Gel electrophoresis of real time products was also performed in order to assess the specificity of the amplification.

## Statistical analysis

The results were expressed as means ± standard deviations (SD). Prior to data analysis, the Kolmogorov–Smirnov test was used to check for normality of distribution. Parameters not showing normal distribution were log-transformed. The Levene’s test was used to test the homogeneity of variance among groups. If homogeneity of variance assumption was violated, Welch test was performed and the respective p value was reported. Differences in data obtained in real-time gene expression experiments, densitometric analysis, and image analysis between controls and cells stimulated with different ligands were analysed using Student’s t-test or ANOVA for comparison of multiple parameters with Bonferroni correction.

## Results

### Expression of TRPV1 in human testis

**Fig 1:**
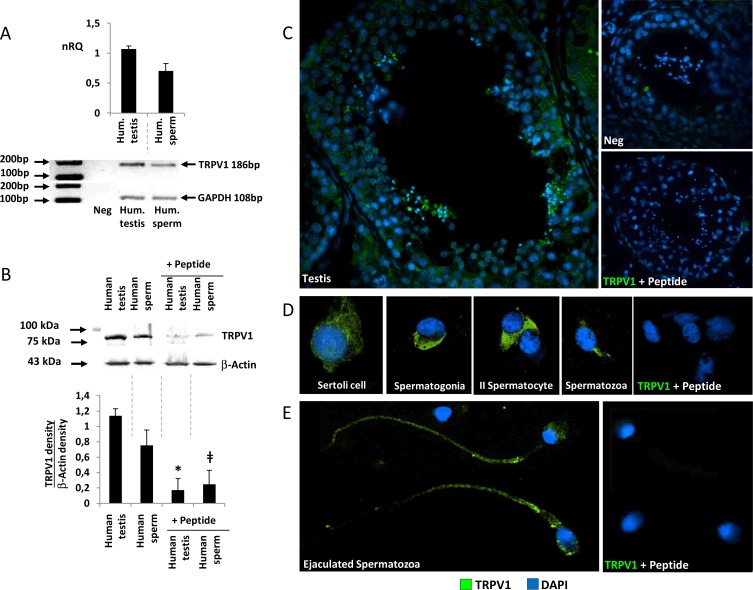
Expression of TRPV1 in human testis. A) Analysis of the expression of *TRPV1* mRNA in human testis biopsies (Hum. testis) and ejaculated human spermatozoa (Hum. sperm) by RT-PCR. Data are reported as normalized relative quantification (nRQ). Relative expression was also reported as gel electrophoresis analysis of the target amplification product at 186 base pairs (*TRPV1*) and *GAPDH* as housekeeping gene (108 base pairs). B) Analysis of the protein expression of TRPV1 in human testis biopsies (Human testis) and ejaculated human spermatozoa (Human sperm) by western blot. The specificity of primary immunoreaction was assessed by co-incubation with the immunogen peptide (Peptide). Relative expression was reported as the ratio between the band density at ~90 kDa (TRPV1) and the band density of β-Actin as housekeeping (43 kDa). Images are representative of six independent experiments. Significance: * = P<0,05 *vs* human testis; ǂ = P<0,05 *vs* human sperm. C) Evaluation by immunofluorescence of TRPV1 protein localization (green) in whole human seminiferous tubule, testis cell populations from fine needle aspiration specimens (D) and ejaculated spermatozoa (E). Samples were counterstained with DAPI (blue). In the negative control (Neg insert in C) primary antibody was omitted. As additional control condition, immunogen peptide was added in the reaction mixture (+ Peptide).

Gene expression of *TRPV1* in human testis was assessed by real time-PCR (Fig 1A). *TRPV1* mRNA was highly expressed in both whole testis biopsies and ejaculated spermatozoa. Gel electrophoresis of amplification products showed the band amplicons at the expected molecular length (186 bp) for both whole testis specimens and samples of ejaculated spermatozoa.

Consistency of gene expression with protein expression was evaluated by western blot analysis (Fig 1B). A specific protein band of nearly 90 kDa was detected for both whole testis and ejaculated sperm specimens. The specificity of the primary immune-reaction was assessed by co-incubation of the primary antibody with the control peptide antigen that significantly reduced the signal intensity.

The expression pattern of TRPV1 was also assessed by immunofluorescence (Fig 1C to 1E). In sections of testis biopsies where morphology and spermatogenesis was preserved, a positive and diffuse staining for TRPV1 was detected for any cell component of the seminiferous tubule (Fig 1C, 1D and 1E). This evidence was confirmed by immunofluorescence performed on specimens from testis fine needle aspiration. Accordingly, both Sertoli cells and all stages of differentiation of germ line cells displayed a positive immune-staining for TRPV1 (Fig 1D). Finally, ejaculated spermatozoa showed positive and diffused expression of TRPV1 at the principal piece of flagellum, the acrosomal region and the midpiece (Fig 1E). Co-incubation with control peptide antigen resulted in sharp blunt of the staining signal (Fig 1S, 1D and 1E).

### Thermotaxis selects sperm cells with higher expression of TRPV1

**Fig 2:**
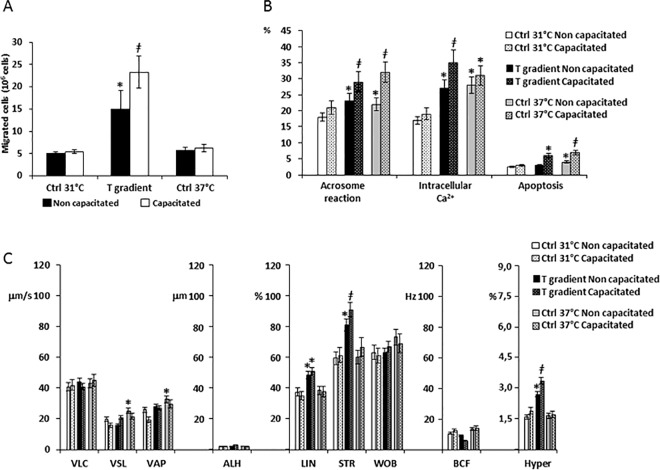
Functional characteristics of sperm cells selected by thermotaxis. A) Quantification of sperm cells selected by thermotaxis in non-capacitated and capacitated conditions. Migration towards a 31–37°C gradient (T gradient), towards 31°-no gradient control (Ctrl 31°C) and towards 37°-no gradient control (Ctrl 37°C) were compared. Data are reported as millions of sperm cells. Significance: * = P<0,05 *vs* both Ctrl 31°C and Ctrl 37°C; ǂ = P<0,05 *vs* corresponding non-capacitated conditions. B) Assessment of sperm acrosome reaction, intracellular calcium levels and apoptosis by flow cytometry. Data are reported respectively as percentage of the CD46-positive (Acrosome reaction), calcium orange positive cells (Intracellular Ca^2+^) and annexin-V-positive cells (Apoptosis) cells migrated towards the T gradient, the Ctrl 31°C or the Ctrl 37°C in either non-capacitated or capacitated conditions. Significance: * = P<0,05 *vs* Ctrl 31°C; ǂ = P<0,05 *vs* corresponding non-capacitated conditions. C) Sperm motility parameters of migrated cells assessed by automated Sperm Class Analyser. Sperm curvilinear velocity (VLC), straight-line (rectilinear) velocity (VSL), average path velocity (VAP), amplitude of lateral head displacement (ALH), linearity (LIN), straightness (STR as VSL/VAP), wobble (WOB as VAP/VLC), beat-cross frequency (BFC), hypermotility (Hyper) were considered in cells migrated towards the T gradient, the Ctrl 31°C or the Ctrl 37°C in either non-capacitated or capacitated conditions. Significance: * = P<0,05 *vs* Ctrl 31°C; ǂ = P<0,05 *vs* corresponding non-capacitated conditions.

The migratory activity, the functional status and the motility parameter of sperm cells undergoing thermotaxis in either non-capacitated conditions or after capacitation was assessed (Fig 2).

As shown in Fig 2A, in absence of capacitation, nearly a three folds higher number of sperm cells migrated towards the T gradient compared with either the Ctrl 31°C or the Ctrl 37°C (respectively 14,9 ± 4,2×10^6^ cells T gradient *vs* 5,1± 0,3×10^6^ cells Ctrl 31°C and 5,71±0,74×10^6^ cells Ctrl 37°C; P = 0,039. Fig 2A). Moreover, capacitation amplified the effect of the T gradient (respectively 23,3±3,66×10^6^ cells T gradient *vs* 5,41± 0,42×10^6^ cells Ctrl 31°C and 6,2±0,82×10^6^ cells Ctrl 37°C; P = 0,027).

The functional status of sperm cells after migration was also assessed (Fig 2B). In non-capacitated conditions, the cells migrated towards the T gradient and Ctrl 37°C showed increased levels of acrosome reaction and intracellular calcium compared to Ctrl 31°C, whilst apoptosis levels were slightly increased in cells migrated towards Ctrl 37°C. Capacitation further and significantly increased these differences, in particular for apoptosis levels in those cells migrated towards the T gradient and in Ctrl 37°C

Motility parameters of the migrated cells are summarized in Fig 2C. In non-capacitated conditions, the cells migrated toward the T gradient showed increased LIN, STR and an increase of the Hyper fraction compared with the Ctrl 31°C. Cells migrated towards Ctrl 37°C had increased values of VSL and VAP. Capacitation was associated to further increase of LIN, STR and Hyper parameters only in those cells migrated towards the T gradient.

**Fig 3:**
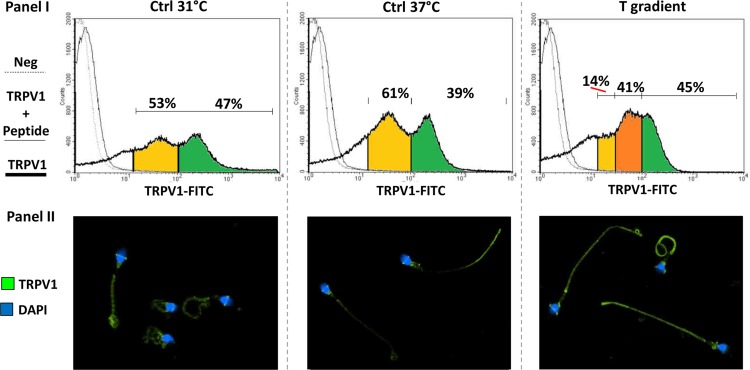
TRPV1 levels in sperm cells selected by thermotaxis. TRPV1 protein expression of the sperm cells migrated towards the 31°-no gradient control (Ctrl 31°C), towards the37°-no gradient control (Ctrl 37°C) and towards a 31–37°C gradient (T gradient). Cells were analyzed by cytometry (Panel I) and immunofluorescence (Panel II). In panel I, histogram plots of cells stained with anti-TRPV1 antibody (thick continuous line), anti-TRPV1 antibody and immunogen peptide (thin continuous line) and negative control with no primary antibody are compared. Anti-TRPV1 antibody cell staining intensity was distinguished within as low (yellow areas), high (green areas) and intermediate (orange areas). In panel II, TRPV1 staining at immunofluorescence appears as green whilst cell nuclei are counterstained with DAPI (blue). Data are representative of six independent experiments.

TRPV1 levels in non-capacitated sperm, assessed by flow cytometry and immunofluorescence, are reported in Fig 3. TRPV1 levels in cells migrated towards Ctrl 31°C (panel I), showed a bi-modal distribution of the staining intensity, clearly identifying two populations of cells respectively with a low staining intensity (yellow area) and a high staining intensity (green area) grossly comprising the 50–60% and 40–60% of cells. No difference in the pattern was observed between Ctrl 31°C and Ctrl 37°C (Fig 3). On the other hand, the cells migrated towards the T gradient showed an enrichment of cells with an intermediate staining intensity (orange area). In particular, the cell population with intermediate and high staining intensity represented the vast majority of cells migrated towards the T gradient (80–85%). The staining pattern of TRPV1, assessed by immunofluorescence (panel II), showed that the cells migrated towards the T gradient had a stronger staining on the principal piece of the flagellum. Capacitation had no major effects on either TRPV1 levels or the expression pattern (data not shown).

### Pharmaceutical modulation of TRPV1 in sperm cells

**Fig 4:**
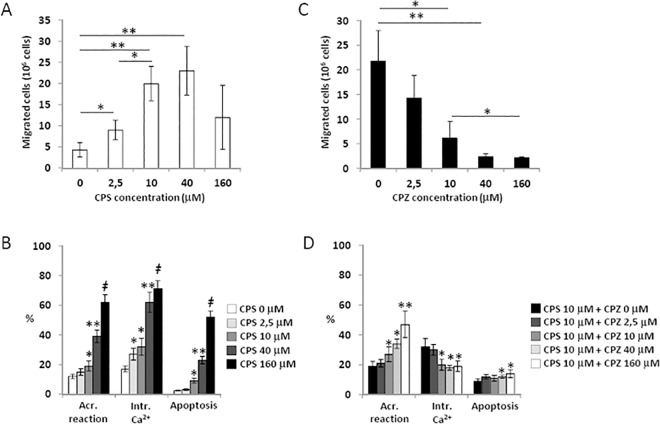
Sperm cells migration under pharmacological modulation of TRPV1. A) Dose-response curve of sperm cells migration towards a capsaicin (CPS) gradient at the concentrations indicated. Significance: * = P<0,05 between the indicated condition; ** = P<0,01 *vs* CPS 0 μM. B) Dose response curve of acrosome reaction (Acr. Reaction), intracellular calcium levels (Intr. Ca^2+^) and apoptosis in sperm cells migrated towards a CPS gradient at the concentrations indicated. Data, obtained by flow cytometry, are reported respectively as percentage of CD46-positive (acr. reaction), calcium orange positive cells (Intr. Ca^2+^) and annexin-V-positive cells (apoptosis). Significance *vs* CPS 0 μM: * = P<0,05; ** = P<0,01; ǂ = P<0,001 C) Dose-response curve of sperm cells migration towards a 10 μM CPS gradient, pre-incubated with capsazepine (CPZ) for 15 minutes at the concentrations indicated. Significance: * = P<0,05 between the indicated conditions; ** = P<0,01 *vs* CPZ 0 μM. D) Dose response curve of acrosome reaction, intracellular calcium levels and apoptosis in sperm cells migrated towards a 10 μM CPS gradient pre-incubated with CPZ for 15 minutes at the concentrations indicated. Data, obtained by flow cytometry, are reported respectively as percentage of CD46-positive (acr. reaction), calcium orange positive cells (intr. Ca^2+^) and annexin-V-positive cells (apoptosis). Significance *vs* CPS 0 μM: * = P<0,05; ** = P<0,01

On the bases of previous results, showing that capacitation essentially amplified the migrating activity of sperm cells in a T gradient, in this session we focused on non-capacitated cells. Sperm cells exposed for 30 minutes to the CPS gradient showed migrating activity whose extent was dependent on the concentration of CPS (Fig 4A). Concentration higher than 40 μM showed no additional effect compared to 10 μM. Secondly, we evaluated the time-dependence of sperm migratory activity towards a 10 μM CPS gradient and found that a plateau was reached after 30 minutes of migration (S1B Fig). Assessment of the functional status of migrated sperm showed a progressive increase of both acrosome reaction and intracellular calcium levels with the increase of CPS concentration (Fig 4B). Most importantly, CPS concentrations higher than 10 μM were associated to relevant levels of apoptosis (>20% of cells), thus migration towards a 10μM CPS gradient for 30 minutes was then assumed as the optimal compromise for the sensitivity of the assay.

We then assessed the effect of pre-incubation with capsazepine (CPZ) on sperm cell migration towards the 10μM CPS gradient for 30 minutes (Fig 4C). CPZ prevented sperm cells migration in a concentration-dependent manner, achieving a plateau at the concentration of 10 μM. In particular, incubation with 10 μM CPZ for periods longer than 15 minutes, resulted in no further inhibition of sperm migration (S1C Fig). On the other hand, sperm cells pre-incubated with CPZ showed a concentration-dependent increase of the acrosomal reaction and a decrease of intracellular calcium levels (Fig 4D). In addition, a slight increase of the percentage of apoptotic cells was observed for concentrations of CPZ 40 and 160 μM. We thus used pre-incubation with 10 μM CPZ for 15 min as the optimal condition for TRPV1 inhibition.

**Fig 5:**
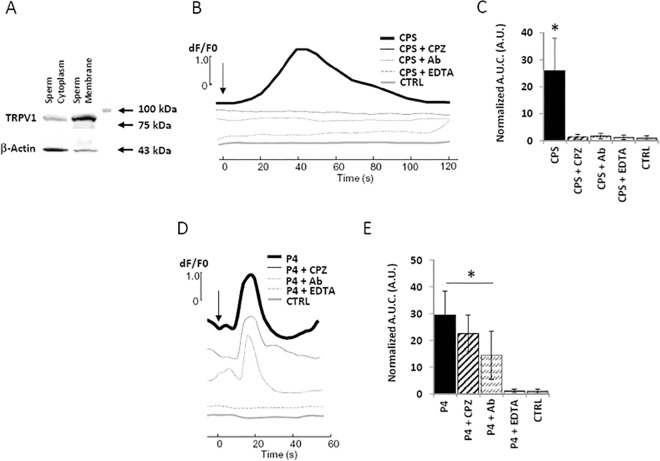
Analysis of TRPV1-dependent calcium trafficking in human spermatozoa. A)Western blot analysis of TRPV1 expression in isolated sperm cytoplasm and sperm membrane obtained by differential centrifugation. β-Actin was used as cytoplasm marker. Images are representative of three independent experiments. D) Dynamic of intracellular calcium concentration in sperm cells stimulated with 10 μM capsaicin (CPS), CPS pre incubated with 10 μM capsazepine (CPS + CPZ), CPS pre incubated with 2,5 μM rabbit polyclonal anti-TRPV1 antibody (CPS + Ab), CPS with chelation of extracellular calcium obtained with addition of 6mM EDTA (CPS + EDTA) compared to unstimulated conditions (CTRL). In C) values of area under the curves normalized on controls (Normalized A.U.C.) are compared. Significance: * = P<0,05 *vs* CTRL B) Dynamic of intracellular calcium concentration in sperm cells stimulated with 10 μg/mL progesterone (P4), P4 pre incubated with 10 μM capsazepine (P4 + CPZ), P4 pre incubated with 2,5 μM rabbit polyclonal anti-TRPV1 antibody (P4 + Ab), P4 with chelation of extracellular calcium obtained with addition of 6mM EDTA (P4 + EDTA) compared to unstimulated conditions (CTRL). In E) values of area under the curves normalized on controls (Normalized A.U.C.) are compared. Significance: * = P<0,05 *vs* CTRL

The dynamic of calcium trafficking associated to TRPV1 stimulation was finally investigated (Fig 5). Firstly, western blot analysis for TRPV1 expression was performed on both sperm plasma membrane and sperm cytoplasm isolated by differential centrifugation [28]. A protein signal corresponding to TRPV1 was mainly associated to isolated sperm membrane, characterized by a low representation of β-actin (Fig 5A). Secondly, intracellular calcium concentration ([Ca^2+^]_i_) was monitored in non-capacitated sperm cells through calcium imaging analysis under varying stimuli. Involvement of voltage gated Ca^2+^ channels was excluded by the addition of Verapamil [17]. Representative profiles of [Ca^2+^]_i_ are reported in Fig 5B. CPS 10 μM was able to induce a transient increase of [Ca^2+^]_i_ that peacked within 40 seconds from stimulus and returned to basal levels within 120 seconds. This effect was not observed when cells were pre incubated with either 10 μM CPZ, 2,5 μM polyclonal rabbit-anti TRPV1 antibody or after chelation of extracellular calcium through the addition of 6 mM EDTA. Representative microscope fields of treated sperm cells, reported as pseudo-colour scale of Fluo-4 AM emission, are reported in S2A Fig. Cumulative [Ca^2+^]_i_ variation, reported as area under the curve (A.U.C.) normalized on control, showed a comprehensive suppression of calcium entry exerted by CPZ, anti-TRPV1 antibody and extracellular calcium chelation. Progesterone (P4) 0,1 μg/ml, used as a reference inducer of [Ca^2+^]_i_ increase [30], was able to induce a faster increase of [Ca^2+^]_i_, peacking in 20 seconds from stimuli (Fig 5D). This effect was inhibited only by extracellular chelation of calcium EDTA. Representative microscope fields are reported in S2B Fig. Analysis of A.U.C. confirmed that neither CPZ nor anti-TRPV1 antibody were effective to suppress the increase of [Ca^2+^]_i_ exerted by P4.

### Blockade of TRPV1 and sperm thermotaxis

**Fig 6:**
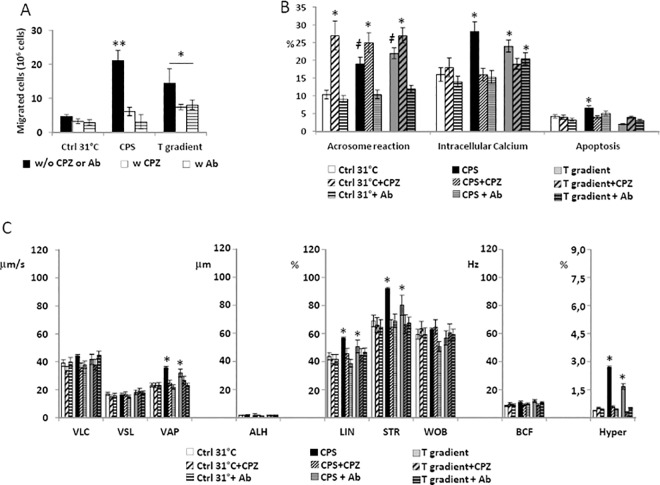
Functional characteristics of sperm cells selected by thermotaxis under pharmacological modulation of TRPV1. A) Quantification of sperm cells migrated towards a 31–37°C temperature gradient (T gradient) or 10 μM gradient of capsaicin (CPS) or towards 31° no gradient control (Ctrl 31°C), without (w/o) or with pre-incubation with 10 μM capsazepine (CPZ) or 2,5 μM rabbit polyclonal anti-TRPV1 antibody (Ab). Data are reported as millions of cells migrated. Significance vs Ctrl 31°C w/o CZP or Ab: * = P<0,05; ** = P<0,01. B) Acrosome reaction, intracellular calcium levels and apoptosis in the sperm migrated towards the T gradient or the CPS gradient or towards Ctrl 31°, without or with pre-incubation with CPZ or Ab. Data, obtained by flow cytometry, are reported respectively as percentage of CD46-positive (acr. reaction), calcium orange positive cells (intr. Ca^2+^) and annexin-V-positive cells (apoptosis). Significance: * = P<0,05 *vs* corresponding condition without CPZ; ǂ = P<0,05 *vs* Ctrl 31°C. C) Sperm motility parameters assessed by automated Sperm Class Analyser (C). Sperm curvilinear velocity (VLC), straight-line (rectilinear) velocity (VSL), average path velocity (VAP), amplitude of lateral head displacement (ALH), linearity (LIN), straightness (STR as VSL/VAP), wobble (WOB as VAP/VLC), beat-cross frequency (BFC), hypermotility (Hyper) were considered in cells migrated towards the T gradient or the CPS gradient or towards Ctrl 31°, without or with pre-incubation with CPZ or Ab. Significance: * = P<0,05 *vs* 31°C.

Non-capacitated sperm cells were allowed to migrate towards the Ctrl 31°C, towards the T gradient and towards the 10 μM CPS gradient, either without or with of pre-incubation with 10 μM CPZ or 2,5 μM polyclonal rabbit-anti TRPV1 antibody for 15 minutes (Fig 6A). Despite pre-incubation with CPZ was associated with a sharp blunt of cell migration in any of the tested conditions, CPZ did not completely abolish migration towards the T gradient. An overlapping trend was observed after pre-incubation with anti TRPV1 antibody.

Parallel evaluation of sperm function (Fig 6B) showed that migration towards both T gradient and the CPS gradient was associated to increased levels of acrosome reaction that showed even further increase after pre-incubation with CPZ. Moreover, both the cells migrated towards the T gradient and those migrated toward the CPS gradient showed increased levels of intracellular calcium, a condition that was essentially reversed by pre-incubation with CPZ. In addition, none of the assessed experimental conditions was associated with increased levels of apoptosis, with the exception of cells migrated towards the CPS gradient that showed a slight but significant increase of apoptosis parameter. Pre incubation with anti TRPV1 antibody was associated with a sharp blunt of acrosome reaction and levels of apoptosis in both migration towards the T gradient and the CPS gradient. However, migration towards the T gradient in presence of anti TRPV1 antibody was not associated to complete blunt of the intracellular calcium increase that appeared slightly but significantly higher than Ctrl 31°C.

Evaluation of motility parameters (Fig 6C) showed that, compared with Ctrl 31°C, both migration towards T gradient and towards CPS gradient were associated with higher VAP, LIN, STR and Hyper values. Pre-incubation of cells with either CPZ or anti TRPV1 antibody essentially abolished those differences.

**Fig 7:**
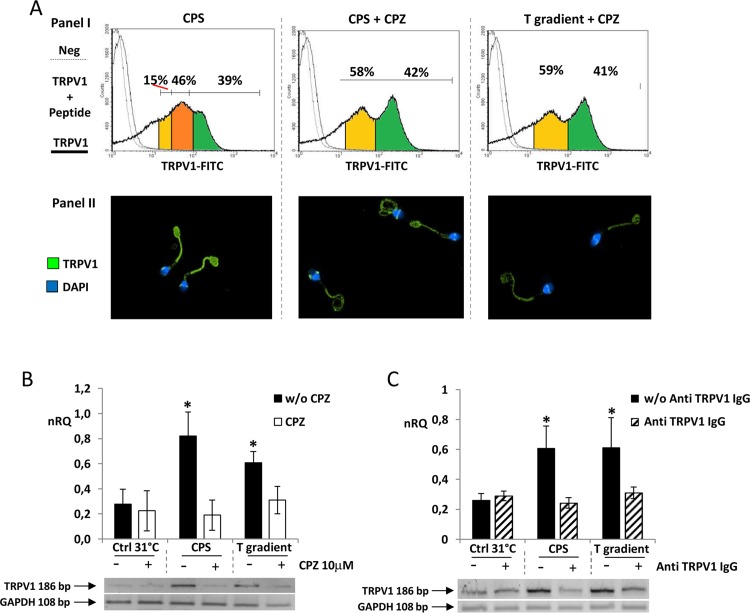
TRPV1 levels in of sperm cells selected by pharmacological modulation of TRPV1. A) TRPV1 protein expression of sperm cells migrated towards a 10 μM CPS gradient (CPS) or towards a 10 μM CPS gradient with pre-incubation with 10 μM CPZ (CPS+CPZ) or towards a 31–37°C temperature gradient with pre-incubation with 10 μM CPZ (T gradient + CPZ). Analysis was performed by flow cytometry (Panel I) and immunofluorescence (Panel II). In panel I, histogram plots of cells stained with anti-TRPV1 antibody (thick continuous line), anti-TRPV1 antibody and immunogen peptide (thin continuous line) and negative control with no primary antibody are compared. Anti-TRPV1 antibody cell staining intensity was distinguished within as low (yellow areas), high (green areas) and intermediate (orange areas). In panel II, TRPV1 staining an immunofluorescence appears as green whilst cell nuclei are counterstained with DAPI (blue). Data are representative of six independent experiments.B-C) RT-PCR analysis of the expression of *TRPV1* mRNA sperm cells migrated migrated towards the T gradient or the CPS gradient or towards the Ctrl 31°, without (w/o) or with pre-incubation with CPZ (panel B) or 2,5 μM rabbit ployclonal anti-TRPV1 antibody (Anti TRPV1 IgG, panel C). Data are reported as normalized relative quantification (nRQ). Relative expression was also reported as gel electrophoresis analysis of the target amplification products at 186 base pairs (*TRPV1*) and *GAPDH* as housekeeping gene (108 base pairs). Significance: * = P<0,05 *vs* Ctrl 31°C

Finally, TRPV1 levels by flow cytometry and immunofluorescence were assessed (Fig 7). Referring to results in Fig 3, sperms migrated towards the CPS gradient showed an enrichment of cells with an intermediate staining intensity (orange area) that, together with the cell population with high staining intensity for TRPV1 (green area), represented the vast majority of the migrated cells (~85%, Fig 7A, panel I). Pre-incubation with CPZ showed no enrichment of the cell population with intermediate staining intensity in either sperms migrated towards the of CPS gradient or the T gradient. Notably, cells migrated towards the CPS were featured by an increase of the TRPV1 staining on the principal piece of the flagellum (Fig 6A, panel II). In addition, cells migrated towards the T gradient or the CPS gradient had higher experssion of *TRPV1* mRNA compared with Ctrl 31°C. Pre incubation with CPZ or anti TRPV1 antibody blunted these differences (Fig 7B and 7C respectively).

## Discussion

Thermotaxis is an acknowledged mechanism accounting for the drive of sperm oriented motility and is likely subtended by a redundancy of signalling pathways. In this manuscript we show a major involvement of TRPV1 in this physiological process.

TRPs are a family of voltage-dependent non-selective cation channels [31] whose member TRPV1 is activated by membrane depolarization [32]. From a mechanistic point of view, the increase in environmental temperature as well as stimulation with CPS, the specific chemical agonist of TRPV1, associates with shifts of its voltage-dependent activation curve toward a lower voltage threshold [33]. The physiological role of TRPV1 in sperm cell has been recently investigated, in particular for its involvement in transmembrane calcium trafficking [17]. Interestingly, it is also an acknowledged molecular target of anandamide, the major endocannabinoid agonist [16, 34]. However, few data are available about the expression of TRPV1 in the cell population of the seminiferous tubule. Here we show that the expression of TRPV1, both in terms of mRNA and protein, is not a confined to the sole sperm cell, but is a shared feature of both germ-line cells, from spermatogonia to spermatocyte, and even of Sertoli cells. This wide expression of TRPV1 within the seminiferous tubule suggests its involvement in the temperature-dependent regulation of spermatogenesis [35, 36]. This hypothesis obviously deserves confirmation in humans and, to this regard, sauna exposure could represent a reliable experimental model to disclose the possible role of TRPV1 in the spermatogenetic process [36].

In this study we investigated the possible involvement of TRPV1 in sperm thermotaxis. To this regard, capacitation has been generally considered a mandatory step to allow migration towards a temperature gradient [9], however a large fraction of non-capacitated spermatozoa has been described to be responsive to thermotactic stimuli [37]. Accordingly, we found that non-capacitated sperms are able to migrate toward 31–37°C gradient and, in agreement with previous studies, migrated cells show an ordered increase of motility parameters [38]. On the other hand, we report that capacitation essentially amplifies the cell response to the thermotactic stimulus. Dealing with a physiological scenario, a small fraction of the ejaculated spermatozoa undergo capacitation through the penetration of cervical mucus, however these sperms are suggested to unlikely reach the fertilization site [39]. Indeed sperm cells achieve the oviduct by active swimming and passive drag by female genital-tract muscular contraction, then they would be able to sense the thermotactic stimulus represented by the temperature gradient existing between the storage site and the fertilization site [6]. Here capacitation, triggered by the oviductal fluid, would then boost sperm motility augmenting thermotaxis in long range migration and chemotaxis in the close proximity of the oocyte [6].

Available studies on the molecular mechanism of sperm thermotaxis subtend a certain degree of redundancy [12]. Here we the involvement of TRPV1 in this process through different lines of evidence. First, migration toward a temperature gradient is able to select sperm cells with higher expression of TRPV1. Second, on one hand sperms migrate toward a gradient of CPS, the specific agonist of TRPV1, in a concentration dependent manner. On the other hand pre-incubation with either CPZ or polyclonal anti-TRPV1 antibody, specific antagonists of TVPV1, abolish this sperm migrating capability in a concentration dependent manner as well. Interestingly, cells selected by the CPS gradient also show higher expression of TRPV1. Third, pre-incubation with either CPZ or polyclonal anti-TRPV1 antibody reduce sperm thermotaxis without completely abolishing. Of note, both antagonists severely reduced intracellular calcium levels of cells migrated toward both temperature gradient and CPS gradient. Moreover, calcium imaging experiments confirmed the involvement of TRPV1 in extracellular calcium trafficking. This evidence well fits with the calcium-mobilizing properties of the TRPV1 ion channel [17, 40] and with the calcium-dependent gain of hypermotility observed in sperm thermotaxis [41]. Interestingly, we observed that the migration of sperm cells toward CPS gradient was associated with a significant increase of sperm apoptosis. Conversely, incubation with CPZ was invariably associated with increased levels of acrosome reaction. These results can be interpreted in the light of available *in vitro* data suggesting the involvement of CPS and CPZ in signaling pathways beyond TRPV1. In fact, it has been reported that long-term exposure of cells to CPS and/or short term stimulation with CPS in the high concentration range of 100 to 300 μM may trigger enzymatic processes, either in the plasma membrane or in the mitochondria of cells, that subsequently lead to cell death [42–44]. In parallel, it is well established that TRPV1 signaling converges with the endocannabinoid system in sperm cells that, in turn, exerts a modulating effect on fertilizing ability and acrosomal loss of human sperm [45, 46]. In particular, it has been reported that incubation with CPZ associates with premature acrosome reaction in human sperm with a consequent reduction of reacted cells after further progesterone stimulation [16]. Indeed, the involvement of TRPV1 in thermotaxis appears to be located in a wider crosstalk of signalling pathways within the sperm cells. In fact, it has been demonstrated that breakdown products of phosphatidyl-inositides through PLC, such as diacyl-glycerol, potentiate TRPV1 activity [47, 48]. Our results would then reinforce the suggested role of PLC in sperm thermotaxis, on one hand through the IP3-dependent release of calcium from intracellular store and on the other hand through the potentiation of TRPV1 signalling [11].

In conclusion, here we demonstrated a role of TRPV1 as mediator of sperm thermotaxis in humans. Beyond the new insight in the comprehension of this physiological process, it may represent a novel molecular target for the development of advanced therapeutic strategies in male infertility and a new tool for the selection of sperm cells.

## Supporting Information

S1 Fig A) Gating strategy of sperm cell analysis by flow cytometry. Sperm cells identified in a morphological plot (Sperm cells) were distinguished in non-viable and viable by propidium iodide-staining (PI^-^ viable cells) that were further assessed for functional status. B) Time-dependence curve of sperm cells migration towards a 10 μM capsaicin (CPS) gradient. Significance: * = P<0,05 between the indicated condition; n.s. = non significant. C) Time-dependence curve of sperm cells incubation with 10 μM capsazepine (CPZ) before undergoing migration towards a 10 μM capsaicin (CPS) gradient. Significance: * = P<0,05 between the indicated condition; n.s. = non significant.
(TIF)

S2 Fig Representative microscope fields of sperm cells stimulated for calcium imaging experiments. Pictures show the staining intensity for calcium complex with Fluo-4 AM as pseudo-color scale from blue (low) to white (high). In panel A, representative fields are reported for cells stimulated with 10 μM/mL capsaicin (CPS), CPS pre incubated with 10 μM capsazepine (CPS + CPZ), CPS pre incubated with 2,5 μM rabbit polyclonal anti-TRPV1 antibody (CPS + Ab), CPS with chelation of extracellular calcium obtained with addition of 6 mM EDTA (CPS + EDTA), after 40 seconds of stimulation In panel B, representative fields are reported for cells stimulated with 10 μg/mL progesterone (P4), P4 pre incubated with 10 μM capsazepine (P4 + CPZ), P4 pre incubated with 2,5 μM rabbit polyclonal anti-TRPV1 antibody (P4 + Ab), P4 with chelation of extracellular calcium obtained with addition of 6mM EDTA (P4 + EDTA), after 20 seconds of stimulation. In control conditions (CTRL) no stimuli were added.
(TIF)
