# Supplementary figures and images for: Heat Sensing Receptor TRPV1 Is a Mediator of Thermotaxis in Human Spermatozoa
Source: PLoS One. 2016 Dec 16;11(12):e0167622. doi: 10.1371/journal.pone.0167622 (PMC5161326; doi:10.1371/journal.pone.0167622)

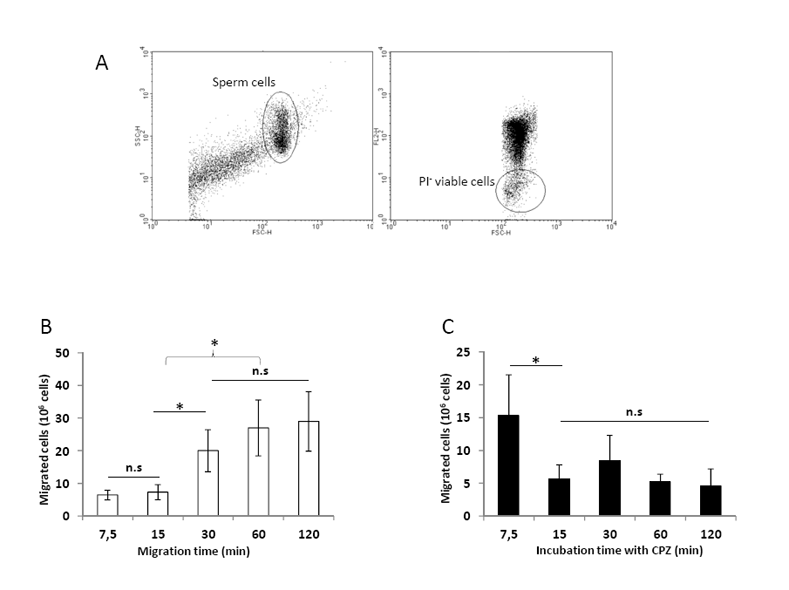

Supplement: S1 Fig — A) Gating strategy of sperm cell analysis by flow cytometry. Sperm cells identified in a morphological plot (Sperm cells) were distinguished in non-viable and viable by propidium iodide-staining (PI- viable cells) that were further assessed for functional status. B) Time-dependence curve of sperm cells migration towards a 10 μM capsaicin (CPS) gradient. Significance: * = P<0,05 between the indicated condition; n.s. = non significant. C) Time-dependence curve of sperm cells incubation with 10 μM capsazepine (CPZ) before undergoing migration towards a 10 μM capsaicin (CPS) gradient. Significance: * = P<0,05 between the indicated condition; n.s. = non significant. (TIF) [file pone.0167622.s001.tif]

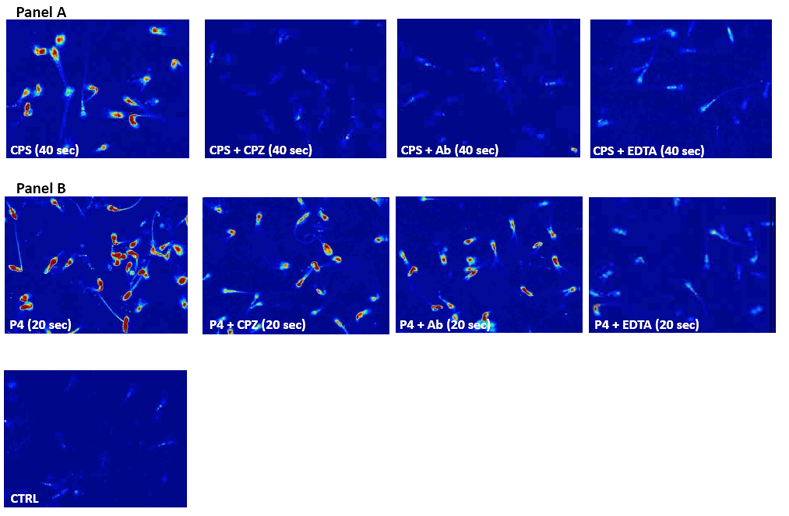

Supplement: S2 Fig — Pictures show the staining intensity for calcium complex with Fluo-4 AM as pseudo-color scale from blue (low) to white (high). In panel A, representative fields are reported for cells stimulated with 10 μM/mL capsaicin (CPS), CPS pre incubated with 10 μM capsazepine (CPS + CPZ), CPS pre incubated with 2,5 μM rabbit polyclonal anti-TRPV1 antibody (CPS + Ab), CPS with chelation of extracellular calcium obtained with addition of 6 mM EDTA (CPS + EDTA), after 40 seconds of stimulation In panel B, representative fields are reported for cells stimulated with 10 μg/mL progesterone (P4), P4 pre incubated with 10 μM capsazepine (P4 + CPZ), P4 pre incubated with 2,5 μM rabbit polyclonal anti-TRPV1 antibody (P4 + Ab), P4 with chelation of extracellular calcium obtained with addition of 6mM EDTA (P4 + EDTA), after 20 seconds of stimulation. In control conditions (CTRL) no stimuli were added. (TIF) [file pone.0167622.s002.tif]
